# Is Self-Determined Motivation a Useful Agent to Overcome Perceived Exercise Barriers in Patients With Type 2 Diabetes Mellitus?

**DOI:** 10.3389/fpsyg.2021.627815

**Published:** 2021-06-18

**Authors:** Heon Jin Kang, John Chee Keng Wang, Stephen Francis Burns, Melvin Khee-Shing Leow

**Affiliations:** ^1^National Institute of Education, Nanyang Technological University, Singapore, Singapore; ^2^Tan Tock Seng Hospital, Singapore, Singapore; ^3^Singapore Institute for Clinical Sciences (A^∗^STAR), Singapore, Singapore; ^4^Lee Kong Chian School of Medicine, Nanyang Technological University, Singapore, Singapore; ^5^Duke-NUS Medical School, Singapore, Singapore

**Keywords:** type 2 diabetes mellitus, self-determined motivation, barriers to physical activity, self-determined theory, physical activity, exercise

## Abstract

*Background*: Devising a program to increase physical activity (PA)/exercise behavior in patients with type 2 diabetes mellitus (T2DM) can meet with limited effectiveness in real-world settings because of the variety of barriers to PA/exercise that individuals need to overcome. An alternative approach is to explore whether targeting motivation as a facilitator may be effective to increase PA/exercise. This study aimed to understand attitudes toward perceived barriers to PA/exercise by examining individual levels of motivation, grounded on self-determination theory, in patients with T2DM. *Methods*: This study used an integrated approach combining qualitative and quantitative analysis. Sixteen patients with T2DM were grouped (*n* = 8 for each group) into either a higher self-motivation (HSM) or lower self-motivation (LSM) group via the Relative Autonomy Index. Thematic and deductive analysis were used to identify attitudes based on ten preconceived barrier themes: apathy, dislike, no priority, lack of support, health problems, lack of knowledge, unfavorable environment, tiredness, lack of time, and financial constraints. Quantitative analysis was to assess statistical differences in the volume of PA/exercise across the two groups, and a mixed-methods analysis was employed to highlight unique cases. *Results*: Patients in the HSM group expressed positive attitudes toward barriers to PA/exercise, while patients in the LSM group expressed a greater degree of hindrance. Although regular PA/exercise is necessary for T2DM management, patients with LSM considered PA/exercise a lesser priority displaying negative attitudes such as apathy and dislike. Conversely, patients with HSM placed greater emphasis on the benefits of PA/exercise regardless of apathy and dislike. Lack of time and health problems were commonly reported in both groups. The volume of PA/exercise corresponded to motivation levels, but there were some unique cases which arose from active commuting habits and severe health problems. *Conclusion*: These findings provide insights on how attitudes to perceived barriers to PA/exercise differ by levels of motivation. One insight was that examining motivation should be an essential consideration when designing practical strategies to overcome PA/exercise barriers in patients with T2DM. Lack of time and health problems exist regardless of motivation levels. Future research requires a tailored approach to managing barriers to PA/exercise in patients with T2DM.

## Introduction

Physical inactivity is a major risk factor for mortality and cardiovascular disease in individuals with type 2 diabetes mellitus (T2DM) (Booth et al., 2017), and regular physical activity (PA)/exercise is crucial in preventing and managing this disease (Dempsey et al., 2014). Despite the importance of regular PA/exercise, individuals with or at risk of T2DM often fail to achieve PA/exercise recommendations (i.e., 150 min of moderate intensity or 75 min of vigorous intensity activity each week) (Morrato et al., 2007; Zhao et al., 2011; Jarvie et al., 2019). Systematic reviews have identified various barriers to PA/exercise (Korkiakangas et al., 2009; Brown et al., 2016). The source of these barriers stems from both internal (e.g., lack of motivation) and external (e.g., financial problems) factors, making it difficult to find efficient solutions to overcome them. Specific barriers related to health concerns such as glycemic control, weight management, symptoms of fatigue, and potential diabetes complications (Casey et al., 2010; Van Dyck et al., 2011) have often been revealed as an impediment to regular PA/exercise as a behavior. Devising a plan that tackles each barrier to PA/exercise might be limited in its effectiveness as individuals often face a variety of perceived barriers, from both internal and external sources, and the combination of barriers could be unique to each individual. Thus attempting to devise a blanket plan that tackles the unique blend of perceived PA/exercise barriers across individuals may be ineffective. An alternative approach for researchers is to explore whether targeting factors that overcome PA/exercise barriers would be more effective in changing behavior in individuals with T2DM. One such factor is motivation.

Motivation can be defined as a psychological force to reinforce action. Grounded in self-determination theory (SDT: Deci and Ryan, 1985, 2002; Ryan and Deci, 2017), motivation can be conceptualized along a continuum by the extent of self-determination from autonomous to controlled. Autonomous motivation, a self-determined form of motivation, includes identified, integrated, and intrinsic regulations. Intrinsic motivation occurs when an individual finds an activity inherently enjoyable. Integrated regulation refers to the process of combining meaningful personal values and identity-relevant commitments. Identified regulation involves volitional actions that are motivated by an appreciation for valued outcomes. Self-determined individuals choose to participate in PA/exercise as they value the activity and derive pleasure and satisfaction from it. Controlled motivation is a less self-determined form of motivation that includes external and introjected regulations. External regulation refers to the satisfaction of external demands, while introjected regulation refers to the internalization of a regulation without accepting it as one’s own. Less-self-determined individuals participate in PA/exercise to gain rewards, to avoid negative consequences, or to avoid feelings of guilt. Possessing self-determined motivation results in the greater likelihood of exhibiting greater effort and perseverance in a behavior (Ryan et al., 2008; Patrick and Williams, 2012; Teixeira et al., 2012; Slovinec et al., 2014; Vancampfort et al., 2015, 2018). To date, research has shown the importance of how the type of motivation associates with PA/exercise behaviors in individuals with T2DM (Sweet et al., 2009; Koponen et al., 2016; Castonguay and Miquelon, 2017; Koponen et al., 2018).

Quantitative research has attempted to link self-determined motivation and barriers to PA/exercise. Barriers are often included as constructs of social cognitive models such as the theory of planned behavior (TPB; Ajzen, 1991) and the social-cognitive theory (SCT; Bandura, 1997). TPB posits intention as the most proximal predictor of behavior. Intention is determined by three TPB social-cognitive variables: attitudes, subjective norms, and perceived behavioral control (PBC). In particular, PBC refers to the extent to which an individual is in control of his or her behavior. In other words, it relates to the barriers and facilitators that they face (Ajzen, 1991). Meanwhile, self-efficacy forms a core construct of SCT, measuring someone’s perceived ability and confidence to perform certain behaviors in given situations. In the context of PA/exercise, self-efficacy measures confidence in overcoming barriers toward PA/exercise.

Studies have integrated both PBC and self-determined motivation (e.g., Fortier et al., 2009; Hagger et al., 2011; Galli et al., 2018). For example, Galli et al. (2018) investigated an older adult population. Their study found a significant and positive effect regarding self-determined motivation on TPB constructs (e.g., PBC) that, in turn, was significantly related to intention. PBC was found to mediate the relationship between motivation and intention. Other research has integrated barrier self-efficacy with self-determined motivation to understand PA/exercise behavior (Thøgersen-Ntoumani and Ntoumanis, 2006; Sweet et al., 2009; Slovinec et al., 2014). One study conducted on patients with coronary heart disease (Slovinec et al., 2014) found that using motivational orientation and barrier self-efficacy interventions for PA/exercise behaviors could alter both short- and long-term PA/exercise adherence. The result showed that both self-determined motivation and barrier self-efficacy were important factors for short-term PA/exercise adherence. Moreover, self-determined motivation remained a significant predictor of long-term PA/exercise adherence. Barrier self-efficacy partially mediated the relationship between motivation and short-term PA/exercise. Sweet et al.’s (2009) longitudinal study found that self-determined motivation mediated the relationship between barrier self-efficacy and PA/exercise in a T2DM population. The study utilized a mediational analysis and found that high barrier self-efficacy could predict 12-month PA/exercise. However, self-determined motivation attenuated the effect, which suggested that their level of self-determined motivation could influence PA/exercise participation.

Although theory-based quantitative research has produced good evidence regarding self-determined motivation and barriers, limited research has been conducted to gain an in-depth understanding of how people with self-determined motivation manage their barriers to PA/exercise and why more self-determined individuals show more positive PA/exercise behaviors compared to less self-determined individuals. A dynamic approach using qualitative methods provides a more comprehensive examination targeting barriers and motivation. For example, Wilcox et al. (2006) qualitative study of arthritis patients examined the attitudes of barriers to PA/exercise based on their PA/exercise levels. The study separated exercisers and non-exercisers and explored differences in barriers, enablers, and motivations for PA/exercise between the two groups. Barriers were classified into four themes: physical, psychological, social, and environmental. Pain, a physical barrier, was the most discussed topic among both groups. Non-exercisers were more likely to quit exercising due to pain, but exercisers displayed a greater likelihood of adapting their PA/exercise and enduring pain to reap benefits associated with the activity. The result suggested that the exercisers realized the benefits of improvements in their symptoms from PA/exercise outweighed the pain, leading to an increased motivation level. The non-exercisers were doubtful of the beneficial effects of PA/exercise and seemed to believe that the physical pain, even if merely passing, may not be worth the potential rewards. This study exemplified how exercisers and non-exercisers possess different attitudes toward their barriers, leading to varying attitudes and approaches to PA/exercise.

Drawing inspiration from an in-depth approach that Wilcox et al. (2006) undertook, examining the attitudes toward barriers to PA/exercise via their motivation levels may give greater insight as opposed to approaching each barrier to PA/exercise individually. Although patients with T2DM may face the same barriers to PA/exercise, there would be differences in their attitudes toward these barriers regarding their motivation levels. By identifying the barriers to PA/exercise faced and their attitudes toward them, researchers could develop more targeted interventions to increase self-determined motivation.

The present study aimed to (1) qualitatively understand the similarities and differences in attitude to barriers concerning the higher and lower levels of self-determined motivation to PA/exercise in patients with T2DM, (2) quantitatively assess statistical differences in the volume of PA/exercise between two groups, and (3) comprehend the reasons behind their PA/exercise behaviors from exceptional such as cases with higher motivation but lower PA/exercise level, or lower motivation but higher PA/exercise level.

## Methods

### Participants

This study obtained the University Institutional Review Board’s approval. A sample of current patients on active surveillance was obtained over a 3-month period from an Endocrine and Diabetes clinic in a restructured tertiary hospital in Singapore after referral from a physician. Patients were selected based on the following criteria: (1) diagnosed with T2DM for at least 6 months, (2) adults (aged ≥ 21 years in Singapore), (3) able to speak and read English, and (4) capable of performing physical movements and not reliant on wheelchairs or crutches. Patients gave their written informed consent, and all interviews were conducted in a quiet setting at the clinic. A total of 20 participants with T2DM were initially recruited.

No specific minimum number of participants is required for in-depth interviews (Brannen and Corm, 2005). Marshall et al. (2013) conducted a meta-analysis of 83 studies published in leading journals and found that the number of participants ranged from 6 to 200. The authors suggested that studies should use 20-30 interviews. Adler and Adler (2012) suggested a sample size of 12, as did Guest et al. (2006). Saunders et al. (2012) found that the acceptable range of the sample size in semi-structured interviews was between 5 and 25. Creswell (2007) said that researchers should expect to undertake between 25 and 30 interviews. However, the real sample size is based on the point of saturation at which no new information or themes are found in the data (Hastings and Perry, 2000). The final sample size of 16 in this study meets this criterion.

Two groups were formed based on the motivation score assessed using the Behavioral Regulation in Exercise Questionnaire-2 (BREQ-2: Markland and Tobin, 2004). The BREQ-2 includes five subscales: intrinsic, identified, introjected, and external regulations as well as amotivation and has been reported to be valid and reliable with a Cronbach’s α of 0.86, 0.73, 0.80, 0.79, 0.83, respectively (Lovell et al., 2016). The BREQ-2 was scored by computing a one-dimensional index of the degree of self-determined motivation, called the Relative Autonomy Index (RAI: Grolnick and Ryan, 1987; Vallerand and Ratelle, 2002).

The RAI is a single score representing the overall degree of self-determination. It is obtained by weighting each behavioral subscale [i.e., amotivation × (−3), external regulation × (−2), introjected regulation × (−1), identified regulation × (+2), intrinsic regulation × (+3)] followed by the summing of these weighted scores. The maximum possible score is 20, and the minimum is −24.

By assigning the mean value from the sum that is the RAI score of the BREQ-2, the two groups were divided into groups of ten each. Four participants whose scores were above and below 2 of the mean score (6.75) were removed from the study to create two distinct groups of participants with clear differences in motivation levels. According to this procedure, one group (*n* = 8) with a mean (standard deviation) RAI score = 1.78 (*SD* = 2.37) was categorized as the ‘lower self-determined motivation (LSM) group,’ and another group (*n* = 8) with a mean RAI score = 14.07 (*SD* = 2.10) was classified as the ‘higher self-determined motivation (HSM) group.’

Supplementary Table 1 shows the demographic details of the 16 participants. The participants within each group had the same gender distribution, similar demographics, no significant differences in age, body mass index (BMI), hemoglobin A1c (HbA1c), and duration of diagnosis of T2DM. The mean BMI for both groups was in the overweight/obese range.

### Procedure and Interview Guide

The protocol consisted of a questionnaire and an interview. The questionnaire comprised questions on demographic information, education level as well as medical information. Self-determined motivation and PA/exercise behavior were measured using the BREQ-2 (Markland and Tobin, 2004) and the Godin Leisure-Time Exercise Questionnaire (GLTEQ: Godin and Shephard, 1985; Godin, 2011), respectively. Participants completed the GLTEQ which assessed the number of times per week an individual performs strenuous (rapid heartbeat, sweating), moderate (not exhausting, light perspiration), and mild (minimal effort, no perspiration) PA/exercise for more than 15 min during their free time. The weekly frequencies of strenuous, moderate, and light activities were multiplied by their respective metabolic equivalent (MET) values of 9, 5, and 3 and then summed to obtain the weekly leisure time score (possible range = 0-119).

After the questionnaire, a semi-structured interview was conducted with each participant. A semi-structured interview was conducted for the following reasons. First, if non-structured interviews were used, PA/exercise barriers would not be understood as intensively. Second, the approach combines components of unstructured and structured interviews and, therefore, has the advantage of both forms of interviews. Interviewees can express their opinions as well as ask the interviewers questions. This approach encourages them to provide more in-depth opinions toward issues raised and easily state their opinions and give reasons for their response. Third, a semi-structured interview provides clear instructions for interviewers, which allows the collection of reliable and comparable qualitative data (Stuckey, 2013). Fourth, it is the most commonly used form of interview used in qualitative research in the diabetes and diabetes self-management field (Patel et al., 2012; Rise et al., 2013).

This study adopted an interview guide based on the self-management framework (Brewer-Lowry et al., 2010; Mathew et al., 2012). The original framework described diabetes self-management as a series of intersections between various tasks (e.g., diet, PA, medications) and resources (e.g., self-care, informal, formal, and medical care). This model presents a clear example of the multi-faceted nature of diabetes self-management. While this model was useful in directing our interview guide, our study was based on PA/exercise behavior and management; thus, interviewees were asked to discuss their experiences with diabetes, PA/exercise behavior, and barriers to PA/exercise.

The interview regarding PA/exercise barriers in this current study was guided by ten themes (i.e., apathy, dislike, no priority, lack of support, health problems, lack of knowledge, unfavorable environment, tiredness, lack of time, and financial constraints) which were derived from a study (Kang et al., 2018) that was established from patients with T2DM in the Singapore context.

Interviews were conducted individually and took approximately 40 min. Each interview was audio-recorded. Detailed field notes were also taken on the spot. The semi-structured interview outline is presented in Supplementary Table 2.

### Data Analysis

An integrated approach using qualitative and quantitative methods was employed. Frist, thematic analysis (Braun and Clarke, 2006) was used to identify, analyze and report themes and sub-themes that emerged from the qualitative data. Deductive approach was used to provide a more detailed analysis of some aspects of the data (i.e., identifying sub-themes), using the preconceived ten themes. Deductive analysis could be useful to identify similarities and differences when one has already established specific research questions to identify the main themes or categories. Once the primary investigator interviewed all of the participants, listened to the audio-recordings and transcribed the interviews, the following phases were carried out: (i) familiarization with the data – two of the other investigators worked on the analysis of the interview transcript and read and re-read each transcript, (ii) coding, (iii) generating initial themes – we used both Nvivo Version 11 (QSR International) and printed copies of the coded data within each preconceived theme to subsequently develop sub-themes, if required, (iv) reviewing themes and sub-themes, and (v) defining and naming themes and sub-themes. For phase (iii), sub-themes were formed inductively without trying to fit into a preexisting coding framework.

Second, a quantitative method using a questionnaire was used to assess statistical differences in the volume of PA/exercise across the HSM and LSM groups. Differences in the number of PA/exercise barriers, RAI, and GLTEQ scores between the LSM and HSM groups were analyzed through a t-test using SPSS version 22.0 (IBM SPSS Statistics).

Then, an integrated analysis combining the interviews and results from the questionnaire was employed to highlight unique cases that had a higher motivation but a low level of PA/exercise or lower motivation but higher PA/exercise level. Despite the mean GLTEQ score of the HSM group being higher than the mean GLTEQ score of the LSM group, an individual from the LSM group had a higher GLTEQ score than the mean score of the HSM group, while two others in the HSM group had a GLTEQ score that was lower than the mean score of the LSM group. These isolated cases showed, that despite their placement in a HSM or LSM group, their level of PA/exercise did not conform to that of peers in their group. Results from the questionnaire in relation to a particular theme are described, followed by excerpts to highlight the meaning of themes from the interviews that align with the questionnaire’s findings.

## Results

### Results From Qualitative and Quantitative Analysis

Supplementary Table 3 presents the barriers to PA/exercise, separated by main and sub-themes, in both groups of patients. The barriers within these ten themes were mentioned more frequently in the LSM group (60 times) than the HSM group (22 times) (*t* = 3.77, *df* = 14, *p* = 0.002). Some sub-themes were derived from some of the main themes: dislike, lack of support, health problems, lack of knowledge, unfavorable environment, tiredness, and lack of time.

**(1) Apathy**

Apathy was the main barrier in the LSM group; however, it was not reported as a barrier to PA/exercise in the HSM group. Six of the eight (75%) LSM interviewees characterized their apathy toward PA/exercise by a lack of energy and desire, with participants often describing themselves as lazy. Conversely, patients with HSM recognized that PA/exercise is a key to their diabetes management. For example, an HSM patient (HSM 2) said, “I think exercise is part of my life to keep me healthy. So I don’t see any lack of willpower.”

**(2) Dislike**

Two sub-themes emerged: (i) discomfort and (ii) negative body image.

**(i) Discomfort**

Half of the patients with LSM (50%) reported that they developed a dislike for PA/exercise due to previous negative PA/exercise experiences, such as feelings of discomfort. These patients felt that PA/exercise was an activity out of their mental and physical comfort zones. One patient (LSM 2) said, “I hate doing exercise. Dislike yes, I hate it. It’s boring. Not stressful, just boring, you know. Uncomfortable, because I sweat. I don’t like that.” A female patient (LSM 5) noted her mental discomfort at exercising in the gym: “I had to stop (my gym)… To go back into it, you know I mean I hate the gym, maybe it’s just me personally I feel like people are judging me sometimes.”

On the other hand, no patients from the HSM group reported a feeling of discomfort toward PA/exercise as a barrier. They were generally more keen, eager, and interested in PA/exercise and had a more positive outlook toward PA, using terms such as “feel better,” “happy,” and “love.”

Patient (HSM 2) said:

“If you ask me to do something that I don’t like, obviously I wouldn’t like to do that activity. But if I love that exercise, for example, swimming, I love swimming very much. So dislike of exercising, it depends on your preference.”

**(ii) Negative body image**

Four (50%) females in the LSM group felt that their bodies were not suitable for specific PAs/exercises such as swimming and expressed embarrassment about their bodies. One patient (LSM 4) mentioned, “I don’t have a good figure, not enough to wear swimming attire.” Another patient (LSM 5) expressed that others would judge her body: “I have a little phobia of wearing the swimming costume in public because I find that I don’t have a good figure. I put on weight, and I am not sexy anymore.”

**(3) No priority**

Most patients from both the LSM and HSM groups recognized that regular PA behavior was important in managing diabetes and was not just a means of assistance to oral medication and nutritious diet. However, an LSM patient (LSM 2) felt that PA/exercise was not helpful and that oral medication was the best treatment for her T2DM. She stated: “Exercise is not necessary to keep blood glucose level lower. It’s not a misconception. It is a true thing because I can tell you that I am better with my testing of my blood sugar with my oral medication [*sic*].”

**(4) Lack of support**

Two sub-themes emerged: (i) preference for an exercise buddy and (ii) limited family support.

**(i) Preference for an exercise buddy**

Five patients (62.5%) in the LSM group, compared to two patients (25%) from the HSM group, preferred PA/exercising with a buddy as they felt this provided additional support and motivation to PA/exercise. These patients expressed that working out alone could sometimes be quite boring; thus, having a buddy made PA/exercise fun.

**(ii) Limited family support**

Four patients (50%) in the LSM group stated that they did not receive support from their families for regular PA/exercise. All four patients were female and felt that the lack of support and understanding from their families prevented them from going outside to PA/exercise. For example, LSM 7 said, “If I go out to PA/exercise, my husband complains to my children.” On the other hand, most patients (87.5%) from the HSM group received enough support from their families to PA/exercise.

**(5) Health problems**

Patients in both groups mentioned health issues as one of the main barriers to PA/exercise. The theme of health problems had two sub-themes.

**(i) Poor physical condition**

Five (62.5%) and three (37.5%) patients in the LSM and HSM groups, respectively, mentioned that their poor physical health condition (e.g., problems with leg, knees, and toes) hampered their regular PA/exercise behavior. “I am very flat-footed, so you know, when you are flat-footed, you don’t have that arch, it hurts sometimes. And the skin thing, and asthma as well. They make exercising very difficult” (LSM 5). “The big problem is my foot and legs. I fall down even during normal walking. Some stairs do not have handles, and my health position gives me a loss of control due to imbalance” (HSM 8).

**(ii) Fear of hypoglycemia**

Hypoglycemia is a diabetes-specific condition. Although hypoglycemia or the fear of hypoglycemia are less common issues for patients with T2DM than with type 1 diabetes mellitus (Brazeau et al., 2008), it can act as a psychological barrier to PA/exercise (Banarer and Cryer, 2004). One interviewee from each group cited their fear of hypoglycemic attacks.

“I am quite fearful of doing exercise because if my sugar level runs down and I lose control, I may not be able to quickly make my way home to eat my meal.” (LSM 4)

“My medical condition. If I over-exercise, I have a lack of sugar which can lead to some other problem.” (HSM 2)

**(6) Lack of knowledge**

Lack of knowledge was divided into two sub-themes; (i) limited information and (ii) knowledge bias as constructs.

**(i) Limited information**

There were differences in the type of PA/exercise engaged by each group. Patients in the LSM group cited cycling as a primary form of PA/exercise. Patients in the HSM group cited swimming, manual activity, jogging or running, and badminton. Patients from both groups also engaged in walking and housework as forms of PA/exercise. Some patients noted that their lack of information limited them in performing PA/exercise.

“I do not have enough knowledge how to exercise properly. I don’t know how much time, or how many days I should exercise.” (LSM 4)

“Right now that kind of exercise behavior that I am doing is very ordinary exercise only. So sometimes I want to have more knowledge on how to exercise better.” (HSM 4)

**(ii) Knowledge bias**

There were cases where participants had their own personal opinion of what constitutes PA. One interviewee in the HSM group misunderstood the definition of PA (i.e., any bodily movement created by skeletal muscles that requires energy expenditure, World Health Organization, 2009) or exercise (i.e., a subset of planned, structured, and repetitive PA and has as a final or an intermediate objective the improvement or maintenance of physical fitness, Caspersen et al., 1985). As a masseuse, she believed that massage was a form of PA/exercise that helps in weight management.

Patient LSM 4 noted that certain types of PA/exercise were discordant with their religious beliefs and thus preferred not to engage in that PA/exercise. Patient LSM 4 said:

“If I do exercise, I want to do yoga, but I cannot take yoga classes because of my Christianity. I am a Christian, so I cannot take yoga. But I like yoga, yes [*sic*].”

**(7) Unfavorable environment**

The unfavorable environment as a barrier to PA/exercise was categorized into two sub-themes: adverse weather conditions and lack of accessibility.

**(i) Adverse weather conditions**

Singapore’s tropical hot and humid climate acted as a barrier to some patients. Six patients (75%) in the LSM group and one (12.5%) patient in the HSM group expressed that the hot temperature and abrupt rain in Singapore prevented them from doing regular PA/exercise. A patient in the LSM group indicated that unexpected weather stopped him from exercising, stating that:

“If it’s raining, I don’t really exercise that day. So I just stay at home. If it rains on the weekend, then I just stay at home.” (LSM 6)

Conversely, most patients with HSM expressed an adaptable and flexible attitude to hot temperatures:

“Weather-wise, it doesn’t matter. If you really want to slim down, you can do it at home also. Not really have to go to the gym outside. [at home] just some stretching, then do some bending, maybe carry some weights.” (HSM 6)

Patient (HSM 2) said:

“Yes. Certain times yes. For example, when you want to go jogging everyday raining, obviously you can’t do jogging. But you can do exercise at home. You can still exercise doesn’t mean that you cannot exercise. You can go and play games indoor games like badminton, table tennis. All these are indoor games.”

**(ii) Lack of accessibility**

Two patients from each group cited the lack of accessibility to PA/exercise facilities as a barrier. For example, one patient in the LSM group who frequently urinates, a symptom of diabetes, refrained from outdoor activities as toilets are not readily accessible.

**(8) Tiredness**

Tiredness had three sub-themes; (i) exhaustion at work, (ii) poor sleep, and (iii) extreme fatigue.

**(i) Exhaustion at work**

Two patients (25%) from each group felt that they were usually too tired to do regular PA/exercise after work. “At times the workload is heavy. Once I go back home, I am tired already” (LSM 8).

**(ii) Poor sleep**

Four patients (50%) from the LSM group mentioned that poor sleep quality made them tired in the day. A patient in LSM admitted that her lack of sleep was due to online gaming at night; the other patient’s sleep was disturbed by frequent urination or being in a noisy environment. Interestingly, no patients in the HSM group highlighted sleep quality as an issue.

**(iii) Extreme fatigue**

Two patients in the LSM group mentioned several times that they were too tired to engage in PA/exercise. Patient (LSM 3) said:

“My body is too exhausted. I do not have the energy to spend to do exercise… Because being exhausted and being tired for the day sometimes makes me feel that it is sufficient it’s good enough reason. Me, to skip my exercise [at the] gym. Physically tired, I am also mentally exhausted.”

One patient with HSM mentioned that fatigue is a kind of special symptom of having high blood glucose. Hence, when she felt tired, she would take a rest first before doing PA/exercise.

“Usually, when I am too tired, I would rest about 1-2 hours. I start doing housework if I am at home…. I climb up the stairs…. I am doing all the things that you need energy to do it. Then you will fight your sugar levels. If you still don’t want to do it, then the sugar level will always be at that level. So it depends on your willpower.” (HSM 3)

**(9) Lack of time**

Lack of time was prominently cited as a barrier in both groups.

**(i) Not enough free time**

Five (62.5%) of the patients in LSM and three (37.5%) in the HSM group stated that they do not have enough free time to doing PA/exercise. Although these patients considered regular PA/exercise to be necessary for diabetes management, they view it as an activity they participate in their free time, rather than a mandatory daily behavior: “My family goes to the gym during the nighttime, I can’t go during at night because I am into online gaming at that time” (LSM 5). Among HSM interviewees, one patient who is a mother of three children emphasized that lack of childcare prevents her from having spare time to do PA/exercise. In contrast, a patient in the HSM group felt that time was not a barrier and that regular PA/exercise was a routine for him. Patient (HSM 5) stated: “Every Saturday morning must exercise. Saturday exercise is a routine.”

**(ii) Demanding time on the job**

Two patients in both groups stated their demanding time on the job is an obstacle. Some younger patients felt that long office hours caused them to be physically inactive. One male patient with LSM who is 32-years-old, working as a teacher, stated: “I do not exercise occasionally because of my tight schedule with work. It is almost a 12-hour shift. When I am at home, I am still doing work.”

**(10) Financial constraints**

Four patients (50%) in the LSM and two (25%) patients in the HSM group mentioned that financial constraints were barriers, as they could not afford access to PA/exercise facilities. An example was, “Sometimes I consider financial barriers. For example, I want to learn swimming, but it costs so much. Maybe I could use the finances to do other stuff” (HSM 4).

### Results From the Quantitative Data Analysis

**FIGURE 1:**
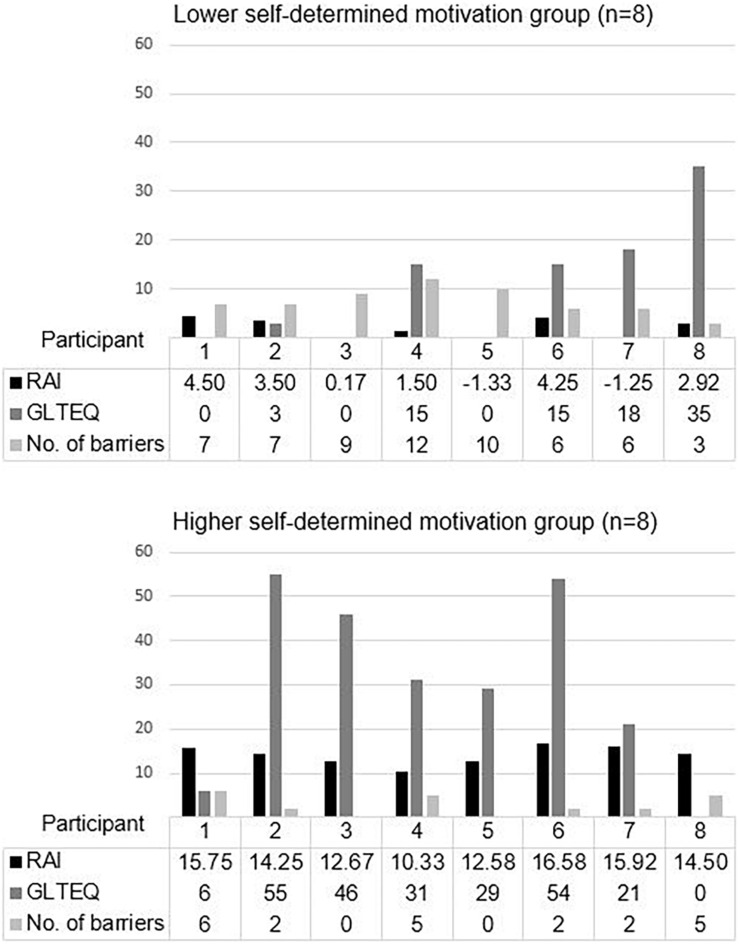
Number of barriers to PA/exercise, self-determined motivation, and a volume of PA/exercise in lower and higher self-determined motivation groups. Relative Autonomy Index; Godin Leisure-Time Exercise Questionnaire; No. of PA/exercise barriers was calculated based on the total number of themes and sub-themes.

Scores for self-determined motivation were calculated from the RAI, while the volume of PA/exercise was calculated from the GLTEQ, and the number of PA/exercise barriers was calculated based on the total number of themes and sub-themes that were found (possible range = 0-18) (see Figure 1). The LSM group showed a mean RAI, GLTEQ score, and PA/exercise barriers of 1.78 (*SD* = 2.37), 10.75 (*SD* = 12.44), and 7.5 (*SD* = 2.78), respectively, while the HSM group showed a mean of 14.07 (*SD* = 2.10), 30.25 (*SD* = 20.76), and 2.75 (*SD* = 2.31), respectively. Independent *t*-tests revealed significant differences between both groups in their self-determined motivation (*t* = 10.98, *df* = 14, *p* = 0.000), PA/exercise behavior (*t* = 2.28 *df* = 14, *p* = 0.039), and PA/exercise barriers (*t* = 3.72, *df* = 14, *p* = 0.002) with the following effect sizes: Hedges’ *g* = 5.49, 1.14 and 1.86 respectively. As the sample size was below 20, we reported the effect sizes by Hedges’ *g* instead of Cohen’s *d* (Kline, 2004).

One patient in the LSM group (GLTEQ score = 35) reported a higher volume of PA/exercise than the average volume in the HSM group (mean GLTEQ score = 30.25). Two patients in the HSM group (GLTEQ score = 6 and 0) reported a lower volume of PA/exercise than the average volume in the LSM group (mean GLTEQ score = 10.75).

**(1) High PA/exercise behavior in the LSM group**

One patient from the LSM group had high volume of PA/exercise (GLTEQ score = 35, RAI score = 2.92). LSM 8, a 56-year-old male, reported three barriers to PA: unfavorable environment (adverse weather conditions), lack of time (demanding time on job), and tiredness (exhaustion at work) – the lowest number among patients with LSM. His high PA/exercise levels came about from commuting to and from work by cycling. Health problems were not a barrier to PA/exercise for him, but he lacked time to perform other forms of PA/exercise aside from commuting to work.

**(2) Low PA/exercise behavior in the HSM group**

HSM 1 and HSM 8 had low GLTEQ scores of 6 and 0, respectively, even though they had high motivation scores of 15.75 and 14.50, respectively. They reported the highest number of barriers among the HSM group – 6 and 5, respectively. HSM 1, a 44-year-old female, had the highest number of barriers (*n* = 6) divided among six sub-themes. These included lack of support (prefer to have an exercise buddy), health problems (poor physical condition), lack of knowledge (limited information), unfavorable environment (lack of accessibility), and lack of time (not enough time, demanding time on job). HSM 8, a 52-year-old female, mentioned barriers with five themes - health problems (poor physical condition), unfavorable environment (lack of accessibility), lack of time (demanding time on job), tiredness (extreme fatigue), and financial constraints. She repeatedly noted that her swollen foot and weak knees hindered normal walking.

## Discussion

The primary finding from the present study reflects that attitudes to barriers differed by motivation level. Patients with HSM were less likely to perceive certain themes as PA/exercise barriers than patients with LSM. Moreover, these differences in reported barriers translated into behavioral differences in the volume of PA/exercise completed within each group. Despite this, certain barriers were common to both groups, and unique cases of patients with incongruent PA/exercise scores from their peers in the same motivational group were also uncovered.

First, there were differing attitudes within the apathy and dislike barriers between the two groups. An apathy toward or a tendency to dislike PA/exercise are concepts related to amotivation, which is defined as the state of lacking the intention to act (Ryan and Deci, 2017). Apathy results from a lack of value for an activity or the belief that the activity will not bring the expected results (Thøgersen-Ntoumani and Ntoumanis, 2006; Ryan and Deci, 2017). Although doing regular PA/exercise is a necessary modification to T2DM management, individuals with low motivation place less value or show no willingness to doing PA/exercise regularly. This may prevent them from participating in regular PA/exercise beneficial to their health. In contrast, individuals with higher motivation expressed their attitudes to barriers about apathy or dislike with characteristics consistent with the various forms of self-determined motivation – identified regulation, integrated regulation, intrinsic motivation (Deci and Ryan, 2002). They valued the benefits associated with PA/exercise (i.e., identified regulation), viewed PA/exercise as congruent with their life goals (i.e., integrated regulation), or partook in PA/exercise for the satisfaction inherent in the activity (i.e., intrinsic motivation).

Particularly the different attitudes showed in negative body image. Under the dislike theme, negative body image emerged only in the LSM group. Body image has been described as the subjective thought on physical appearance, and it can either be positive or negative (Forrest and Stuhldreher, 2007). Negative body image can lead to social physique anxiety, where individuals perceive their bodies to be undesirable and are unwilling to put themselves in situations where their bodies may be assessed negatively (Focht and Hausenblas, 2004). In this current study, female LSM interviewees expressed shame and anxiety when wearing swimsuits or when at the gym, especially when they felt that others were judging their bodies. Though the HSM group had a similar number of female participants and were not significantly different in terms of BMI, negative body image was not found to be a barrier. This suggests that lower motivation toward PA/exercise was associated with negative body image, resulting in hindered PA/exercise behavior. Conversely, the opposite might be possible as well, where having a negative body image may lead to lower motivation levels.

A second emphasis can be regarding poor sleep, a sub-theme of the tiredness barrier. This barrier was only noted in the LSM group, with 50% of the group reporting it, whereas no one in the HSM group highlighted it. Whether poor sleep acts as a barrier to PA/exercise or whether PA/exercise improves sleep quality was uncertain. For the HSM group, regular PA/exercise may have helped to improve sleep quality. Conversely, for the LSM group, not doing PA/exercise regularly could result in poorer sleep quality. A meta-analytic review of studies of adults aged 40 years and above with sleeping problems supported this explanation (Yang et al., 2012), which revealed that regular PA/exercise significantly improves sleep quality. Those who did PA/exercise more frequently noted that they experienced a reduction in time taken to fall asleep and used less medication to counter insomnia.

Some barriers were commented on in both groups. A lack of time and health problems were common to both HSM and LSM individuals. Lack of time was a commonly cited barrier regardless of age, gender, ethnicity, and health status (Trost et al., 2002). Concerning health issues, the nature of T2DM increases the chances of co-morbidities that may impair PA/exercise participation, such as macrovascular complications (Litwak et al., 2013). As T2DM is commonly diagnosed later in life, the body is also often subjected to other ailments like arthritis, which can hinder PA/exercise (Piva et al., 2015). Thus, health problems were a barrier that actively prevented individuals in both groups from engaging in regular PA/exercise beneficial to their condition. Moreover, it could lead to a self-perpetuating cycle of poor PA/exercise behavior as decreased PA/exercise levels could lead to other health issues, such as increased body weight, which further hamper PA/exercise participation. Future research may wish to delve further into looking for effective solutions to break this self-perpetuating cycle. Perhaps from a medical and physical therapy standpoint, there could be an effective protocol to gradually and safely introduce a patient to PA/exercise that is not contra-indicated to their ailments.

Based on the results of the integrated-methods approach, in general, the HSM group reported a higher volume of PA/exercise than the LSM group. However, there were exceptions, with some patients recording a lower motivation level but high volume of PA/exercise, and others higher motivation levels but low volume of PA/exercise. The only case of lower motivation coinciding with a higher volume PA/exercise was a patient engaging in cycling to save on transportation costs. The patient had a lower number of barriers to PA/exercise (*n* = 3) compared with the overall mean reported by LSM group members (*n* = 7.5). A previous study (Jones and Ogilvie, 2012) showed that individuals were motivated by the convenience, speed, cost, and reliability of active commuting rather than its health benefits. Therefore, to ensure that more patients with T2DM participate in active commuting, it is essential to impart knowledge and strategies to help them understand its benefits. An example would be encouraging patients to alight a stop before their intended destination, allowing them to participate in short bouts of PA/exercise that contribute to the total daily volume.

Two patients showed higher motivation but a lower volume of PA/exercise and a relatively higher number of barriers. Both patients cited lack of time, lack of accessibility to PA/exercise, and health problems (i.e., a poor physical condition relating to foot, knee, or leg problems) as barriers to PA/exercise. Both patients stated that they would stop brisk walking when they felt pain, despite a strong desire for PA/exercise. For them, their existing health problems were an obstacle in terms of their PA/exercise choices. This suggests that perhaps consultation with medical or physical therapy professionals would assist in overcoming patients’ physical limitations through eliminating or reducing the pain felt from engaging in PA/exercise.

This research had some limitations. First, PA/exercise was measured based on self-reports instead of direct measures via pedometers and accelerometers, even though the latter provide more precise estimates of energy expenditure and reduce various issues of recall and response bias (Prince et al., 2008). Future studies should measure both direct and self-reported measurements for better reliability.

Second, the measurement instrument used, which was BREQ-2, has limitations. BREQ-2 was initially utilized for this study instead of the more recent BREQ-3 (Wilson et al., 2006) because previous literature supported BREQ-2 (Rosa et al., 2015; Vancampfort et al., 2015; Lovell et al., 2016) and because integrated motivation was not a consideration for this study. However, Wilson et al. (2006) found that integrated regulation was an important tenet of SDT for middle-aged adults as they are likely to develop a sense of integrated regulation for PA/exercise. Furthermore, the use of RAI has been found to lead to some loss of information in research data based on SDT (Wilson et al., 2012). Future research should consider demographics when selecting the measurement instrument to use and the implications of data loss when utilizing RAI scores to evaluate autonomous motivation.

Last, the semi-structured interview questions were designed to elicit responses from the participants based on pre-established barriers instead of asking the participants about their perceived barriers in general. This approach could have resulted in other non-established barriers faced by the participants to be missed during the data collection and analysis process and prevent new categories from emerging. Future studies should consider asking about barriers in general and later go more in-depth to the specific barriers during the interview process.

### Implications for Practice

Self-determination theory is a good theory platform for health promotion programs in patients with T2DM or populations at risk of T2DM. One worksite SDT based intervention program (Pedersen et al., 2018) is a good example here. This intervention aimed to (1) investigate how the worksite and community of works can be incorporated in an intervention designed to move participants toward autonomous motivation for behavioral change, and the effects of such regular PA/exercise on cardiovascular fitness as well as enhancement in health (i.e., reduced blood pressure, waist circumference, and improved cholesterol levels), and (2) test the SDT model of health behavior change.

Participants from a population of employees working within transport and distribution (*n* = 202) were cluster randomized. The participants in those areas were chosen because they were exposed to structural barriers to participation in PA/exercise like shift work, time pressure, and productivity demands.

The 16-week group-based worksite intervention mainly consisted of workshops and PA/exercise support group meetings, and was designed based on the tenets of SDT combined with techniques using motivational interviewing. SDT posits that individuals will develop autonomous motivation when they are supported in their basic psychological needs; autonomy (i.e., feeling a sense of volition and self-endorsed), competence (i.e., feeling effective), and relatedness (i.e., feeling of belonging and being cared for). The results from the program indicated that offering needs-supportive interventions to enhance autonomous motivation resulted in enhancement of cardiovascular fitness and positive changes in health.

With this SDT-based program as an example, patients with T2DM or populations at risk of T2DM could have a good chance improving their attitudes toward their barriers to PA/exercise, increasing their PA/exercise behavior, and gain positive changes in their health behaviors post-intervention. Thus, we propose that it is important to help adults at high risk of, or already diagnosed with, T2DM to identify their barriers to exercise and for them to learn to overcome those barriers. In order to achieve this, the contents of and methods of counseling would need to be developed to create a positive climate that supports the patient’s needs. Further research in this area is important to improve and enhance counseling methods.

## Conclusion

The present study provided some understanding of the differences in attitudes to barriers between patients with T2DM with lower and higher self-determined motivation levels. The results showed that patients with HSM are better at overcoming and managing their barriers to PA/exercise, while patients with LSM listed a wider variety of barriers to PA/exercise. However, some barriers like health problems and lack of time are common to all patients with T2DM irrespective of motivation level. Greater awareness of the specific barriers that plague individuals with T2DM would allow health professionals to understand their concerns better. Additionally, with self-determined motivation as a good predictor of PA/exercise behavior, strategies on improving PA/exercise behavior should focus on how self-determined motivation can be exploited and used to greater advantage. The design of health programs can cater to improving self-determined motivation, helping individuals with T2DM explore more viable PA/exercise possibilities to overcome barriers, and increasing PA/exercise behavior.

## Data Availability Statement

The raw data supporting the conclusions of this article will be made available by the authors, without undue reservation.

## Ethics Statement

The studies involving human participants were reviewed and approved by the Nanyang Technological University Institutional Review Board (#IRB NO 2015-04-005). The patients/participants provided their written informed consent to participate in this study. Written informed consent was obtained from the individual(s) for the publication of any potentially identifiable images or data included in this article.

## Author Contributions

HK and JW conceptualized the study. HK, JW, and SB contributed to the design and implementation of the research and took their roles to the analysis of this research and undertook responsibility of its integrity. ML helped to recruit patients with T2DM and contributed to data collection in the hospital setting. HK was the guarantor of this work and, as such, had full access to all the data in the study and takes responsibility for the integrity of the data and the accuracy of the data analysis. All authors contributed to the article and approved the submitted version.

## Conflict of Interest

The authors declare that the research was conducted in the absence of any commercial or financial relationships that could be construed as a potential conflict of interest.

## Abbreviations

GLTEQ: Godin Leisure-Time Exercise Questionnaire
HSM: higher self-motivation
LSM: lower self-motivation
RAI: Relative Autonomy Index
SDT: self-determination theory
T2DM: type 2 diabetes mellitus.
