# Supplementary material for: Is Self-Determined Motivation a Useful Agent to Overcome Perceived Exercise Barriers in Patients With Type 2 Diabetes Mellitus?
Source: Front Psychol. 2021 Jun 18;12:627815. doi: 10.3389/fpsyg.2021.627815 (PMC8253052; doi:10.3389/fpsyg.2021.627815)
Supplement: Supplementary file 1 [file Data_Sheet_1.docx]

**APPENDICES**

**TABLE 1** Demographic and physical characteristics of patients in two groups

| Variable | LSM (n = 8) | HSM (n = 8) | *P*-value for difference |
| --- | --- | --- | --- |
| Female | 5 | 5 |  |
| Male | 3 | 3 |  |
| Age (years) | 46.25 (SD=12.28) | 49.88 (SD=10.34) | .533 |
| BMI (kg/m^2^) | 29.68 (SD=5.51) | 32.54 (SD=5.66) | .323 |
| HbA1c (%) | 9.38 (SD=4.81) | 7.64 (SD=1.77) | .358 |
| Duration of T2DM (years) | 10.35 (SD=8.63) | 11.25 (SD=9.56) | .846 |

Note. LSM=Lower self-determined motivation; HSM=Higher self-determined motivation; Values are presented as mean and standard deviation; BMI=Body mass index; HbA1c=Hemoglobin A1c.

**TABLE 2** Interview outline

| Briefly introduce yourself.  How do you manage your diabetes? (Diet, PA/exercise behavior, and medication) |
| --- |
| Tell me about your PA/exercise behavior.  How often / How long / PA/exercise routine / What do you do?  Any PA/exercises that you want to do? |
| There are ten PA/exercise barriers listed as below.   1. Apathy 2. Dislike 3. No priority 4. Lack of support 5. Health problems 6. Lack of knowledge 7. Unfavorable environment 8. Tiredness 9. Lack of time 10. Financial constraints   Could you tell me your thoughts about each barrier?  Do you feel that you did not PA/exercise because of?  Why do you feel that each barrier prevents you from PA/exercising? |
| How do you motivate yourself to PA/exercise, although you find it difficult to do so? |

**TABLE 3** Main and sub-themes of PA/exercise barriers between groups

| Main themes | Sub-themes | LSM (n = 8) | HSM (n = 8) |
| --- | --- | --- | --- |
| 1. Apathy |  | 6 | 0 |
| 2. Dislike |  |  |  |
|  | Discomfort | 4 | 0 |
|  | Negative body image | 4 | 0 |
| 3. No priority |  | 1 | 0 |
| 4. Lack of support |  |  |  |
|  | Preference for an exercise buddy | 5 | 2 |
|  | Limited family support | 4 | 1 |
| 5. Health problems |  |  |  |
|  | Poor physical condition | 5 | 3 |
|  | Fear of hypoglycemia | 1 | 1 |
| 6. Lack of knowledge |  |  |  |
|  | Limited information | 2 | 1 |
|  | Knowledge bias | 1 | 1 |
| 7. Unfavorable environment |  |  |  |
|  | Adverse weather conditions | 6 | 1 |
|  | Lack of accessibility | 2 | 2 |
| 8. Tiredness |  |  |  |
|  | Exhaustion at work | 2 | 2 |
|  | Poor sleep | 4 | 0 |
|  | Extreme fatigue | 2 | 1 |
| 9. Lack of time |  |  |  |
|  | Not enough free time | 5 | 3 |
|  | Demanding time on job | 2 | 2 |
| 10. Financial constraints |  | 4 | 2 |
| Total |  | 60 | 22 |

Note. LSM=Lower self-determined motivation; HSM=Higher self-determined motivation.
